# Effect of Oral Care in a Patient with Depression and Burning Mouth Syndrome during the COVID-19 Pandemic

**DOI:** 10.1155/2021/3039269

**Published:** 2021-10-26

**Authors:** Yumiko Nagao, Hitomi Nakagaki, Masahide Tsuji

**Affiliations:** ^1^Department of Public Health, Graduate School of Medicine, Juntendo University, Bunkyo-ku, Tokyo 113-8421, Japan; ^2^Tsuji Dental & Oral Surgery Clinic, Shiragane Machi, Omuta, Fukuoka 836-0052, Japan

## Abstract

Burning mouth syndrome (BMS) is a burning sensation that occurs in the mouth without any underlying cause. There is no satisfactory treatment for BMS, so far. Herein, we report the case of a 74-year-old female with untreated depression who presented with BMS. Despite taking antidepressants, she developed suicidal thoughts, particularly due to the increasing number of coronavirus disease 2019 (COVID-19) cases and suicides in Japan. The symptoms of BMS and the oral discomfort were eliminated using a multifaceted approach, which included the following: continuous application of the oral care gel “REFRECARE-H®” to the mucous membranes, regular dental visits, collaboration with medical and dental professionals, and administration of zinc preparations. Her suicidal thoughts had disappeared, and her quality of life, assessed using the visual analogue scale, was improved following the treatment. Dentists should strive to provide oral care, while providing treatment in collaboration with specialists, for the early detection of depression and zinc deficiency in patients with BMS.

## 1. Introduction

The International Association for the Study of Pain (IASP) described burning mouth syndrome (BMS) as a chronic disease characterized by a burning sensation in the oral mucosa for which the cause cannot be identified [1]. In 2018, the IASP defined the diagnostic criteria for BMS as follows [2]: (a) oral pain fulfilling criteria (b) and (c); (b) recurring daily for >2 hours/day for >3 months; (c) pain has both of the following characteristics: (1) burning quality and (2) felt superficially in the oral mucosa; (d) oral mucosa is of normal appearance and clinical examination including sensory testing is normal; and (e) not better accounted for by another diagnosis.

The burning pain is often accompanied by a tingling sensation, numbness, and dryness in the mouth [3–6]. The prevalence of BMS in the general population is estimated to range from 0.7% to 15%, with a male-to-female ratio of 1 : 7; women are more affected during the peri- and postmenopausal periods [7, 8]. The etiology of BMS is unclear, but it has been suggested that it has a neuropathic origin involving the central and peripheral nerves [9, 10]. According to Feller et al., the pathogenesis of BMS is complex because it involves several psychogenic factors and dysregulated peripheral and central pain pathways [11]. BMS can be treated with clonazepam, a medication used to treat neuropathic pain [12]; vitamin, zinc, and hormone replacement therapy [13–15]; and behavioral cognitive therapy [16].

On the other hand, according to the Ministry of Health, Labor, and Welfare, the number of suicides in Japan in 2020 had increased by 4.5% (*n* = 912) compared to the previous year, marking the first increase in 11 years [17]. While the number of male suicides had decreased for the 11^th^ consecutive year to 14,055, the number of female suicides had increased to 7,026. The Ministry of Health, Labor, and Welfare has indicated that the increase in the number of female suicides might be due to the worsening of factors such as domestic violence (DV) under the extraordinary circumstances of the severe acute respiratory syndrome coronavirus 2 (SARS-CoV-2) epidemic. Tanaka and Okamoto reported that effective prevention of suicide among vulnerable populations is an important public health issue in response to the increase in suicide rates among women and children and adolescents during the COVID19 pandemic [18].

Previously, we reported that the use of REFRECARE-H® (EN Otsuka Pharmaceutical Co. Ltd., Tokyo, Japan), a hinokitiol-containing oral care gel, improved the symptoms of BMS [19] and the quality of life (QOL) of patients with oral lichen planus [20]. Herein, we report a case of a 74-year-old woman who developed suicidal thoughts during treatment for BMS and was treated with REFRECARE-H® in collaboration with other medical professionals.

## 2. Case Presentation

In September 2019, a 74-year-old Japanese woman visited the Tsuji Dental and Oral Surgery Clinic (Fukuoka, Japan) complaining of a tingling pain and dryness in the tongue and a salty taste in the mouth when at rest; the symptoms had begun about five to six years ago (2013–2014). She had frequently consulted her family physician and dentist before visiting our clinic but did not receive any proper advice or treatment for the symptoms. No organic abnormalities were observed in the oral cavity. Evaluation of the tongue dryness using an oral moisture-checking device (Mucus®; Life Co. Ltd., Saitama, Japan) [21] resulted in normal values (30.1). In the taste test using the salt-impregnated test paper, SALSAVE® (Advantec Toyo Co. Ltd., Tokyo, Japan), which was prepared with various salt concentrations (0.6, 0.8, 1.0, 1.2, 1.4, and 1.6 mg/cm^2^), she was unable to distinguish the saltiness, even at high concentrations. The patient was not in the habit of drinking or smoking. No signs of gingival inflammation, oral lichen planus, Sjögren's syndrome, *Candida albicans*, or oral cancer were found in the mouth. Serum anti-SS-A and anti-SS-B antibodies were negative, and there were no clinical signs of Candidiasis which was also confirmed with mycological swab and fungal culture. Subsequently, the patient was diagnosed with BMS by an oral surgeon at our clinic.  Table 1 shows the medications that the patient was taking at the time of the first visit to our clinic. Some of these drugs had been reported to have oral side effects, but the drugs could not be systemically changed or discontinued.

She had developed depression, hypertension, angina pectoris, and idiopathic atrial fibrillation about 25 years ago (at the age of 50), cervical and lumbar stenosis at the age of 55, and reflux esophagitis at the age of 65, for which she was treated by various specialists. Her depression was triggered by the illness and death of her mother when she was 50 years old and later exacerbated by the illness and death of her son when she was 57 years old. The depression was left untreated since February 2019 due to relocation. Furthermore, the patient had undergone cholecystectomy for a benign gallbladder tumor at the age of 39 and an endoscopic polypectomy for colon cancer at 66 years of age.

The pathogenesis of BMS was explained to the patient in detail. She was instructed to brush her teeth after meals and apply REFRECARE-H® to the oral mucosa. Additionally, preventive treatment for periodontal disease and denture prosthesis for the missing teeth were provided at the clinic. The patient had not been treated for depression for over six months; hence, she was referred to a psychiatric clinic and started on antidepressants. In October 2019, the following three medications were administered by the psychiatrist: escitalopram oxalate, a selective serotonin reuptake inhibitor (SSRI), triazolam, and flunitrazepam as benzodiazepine.

**Figure 1:**
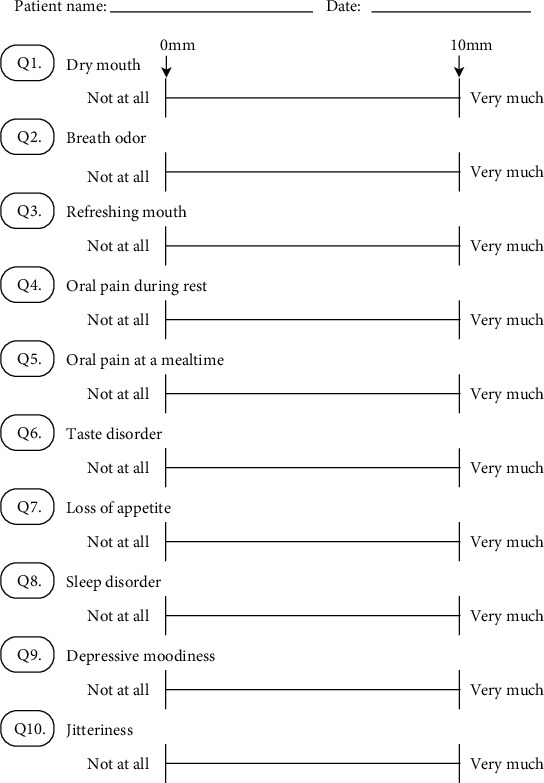
The VAS of 10 items. The VAS is a horizontal line, 100 mm in length, anchored by word descriptors at each end.

**Figure 2:**
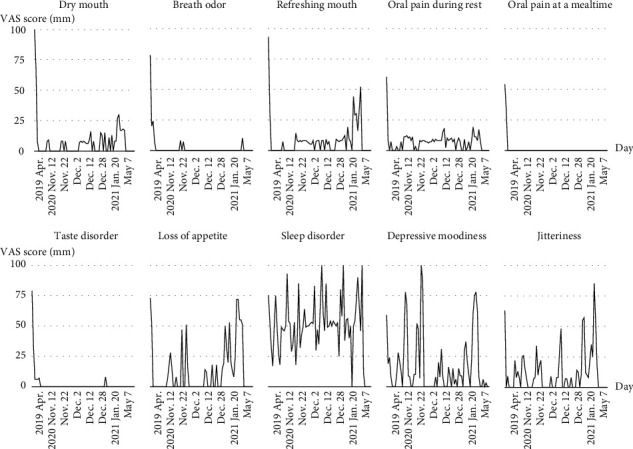
The distributions of the VAS scores before the dental visit and during the application of REFRECARE-H.

The visual analogue scale (VAS) is a simple and frequently used method to evaluate variations in pain intensity [22]. It is represented by a horizontal line, 100 mm in length, anchored by word descriptors at each end, as illustrated in Figure 1; the patients marked the point that they felt most accurately represented their perception of their current state. The VAS score was determined by measuring in mm from the left end of the line to the point that was marked by the patient. The VAS was measured 5 months prior to the patient's visit to our clinic (April 2019) and periodically between September 6, 2019, when the patient first visited our clinic, and May 21, 2021. The mean and changes in the score are shown in Table 2 and Figure 2, respectively. Application of REFRECARE-H® improved the QOL such as dry mouth, breath odor, refreshing mouth, oral pain during rest, oral pain at a mealtime, taste disorder, loss of appetite, sleep disorder, depressive moodiness, and jitteriness.

In October 2019, the patient's tongue pain symptoms had decreased and regular monthly follow-up showed an improving trend. However, since the beginning of 2020, following the prevalence of SARS-CoV-2 infection in the country, a nationwide state of emergency was declared in April and news of the suicides of several celebrities was reported in July. Consequently, she began to admit to suicidal ideation by August 2020, saying “I want to die too” every time she had a dental revisit. Therefore, the frequency of visits to our clinic was increased, information was shared with the psychiatrist and gastroenterologist, and oral care was implemented through medical cooperation. At each visit, we, the dentists, made sure that there were no physical problems in the oral cavity or head and neck areas and explained these to the patient, as well as instructed her to eat, sleep, and exercise regularly. We also listened to and supported the psychosocial issues behind the patient's symptoms. At each visit, we displayed the VAS score visualized over time and explained the clear improvement in BMS and depressive symptoms compared to that before treatment. The application of REFRECARE-H® to the oral mucosa was continued daily. Eventually, the suicidal thoughts disappeared in December 2020. On December 26, she suffered a thoracic vertebrae fracture in a traffic accident and was required to undergo rest therapy but she never complained of wanting to die. In March 2021, the symptoms of BMS completely disappeared and have not recurred since.

From the initial visit to the present, the results of the tongue dryness assessments were normal. However, in the taste test using SALSAVE®, she was still unable to distinguish the saltiness in December 2020. Subsequently, about two months later (February 2021), she was able to recognize the taste naturally (value, 1.4), and in March, she could recognize the saltiness at low concentrations (value, 0.8). She presented with a low serum zinc level (67 *μ*g/dL) on March 26, 2021 (Table 3), and was treated with zinc acetate dihydrate (NOBELZIN®, Nobelpharma Co. Ltd., Tokyo, Japan) in April. The normal range of serum zinc concentration is from 80 *μ*g/dL to 130 *μ*g/dL. However, oral administration of NOBLEZIN was discontinued after 3 weeks due to adverse drug reactions such as diarrhea and itching. On May 7, the serum zinc level had been reduced to 70 *μ*g/dL (below normal) but the taste sensitivity remained improved and she was able to recognize low concentrations of saltiness (value, 0.6). As shown in Table 3, free thyroxine 4 (FT4), thyroid-stimulating hormone (TSH), HbA1c, and blood glucose levels were normal, indicating no abnormal thyroid function or diabetes mellitus in this patient.

## 3. Discussion

The tongue pain symptoms in this case showed a tendency to improve after SSRI and benzodiazepines were administered to treat comorbid depression, indicating that BMS may be due to psychological factors. As for the zinc deficiency, since no blood test was performed at the time of the first visit, it is impossible to evaluate whether the patient had zinc deficiency from the time of the first visit or from March 2021. However, since the patient later showed improvement in taste disorder even though the zinc level was below normal, it is considered that the underlying cause of the painful tongue symptoms was not zinc deficiency. In other words, both tongue pain and taste disorder have a great possibility of being a series of symptoms associated with depression. Four of the medications (irbesartan and amlodipine besilate, bepridil hydrochloride hydrate, dabigatran etexilate methanesulfonate, and limaprost alfadex) that the patient was taking when she was diagnosed with BMS have side effects of oropharyngeal discomfort, dry mouth, and taste disorders as shown in Table 1. However, even though these medications are still being taken, the oral symptoms have disappeared, and therefore, the possibility of drug side effects is very low.

The diagnosis of BMS is defined by IASP as “can only be made when the oral mucosa is clinically normal and all systemic and local causes for a burning sensation have been ruled out” [11]. Because it is important to examine systemic and local causes of burning sensations in the mouth, blood tests to screen for comorbidities and other chronic pain should be performed in addition to performing a basic oral examination at the initial visit.

BMS patients often complain of taste abnormalities [23–25]. It has been reported that 69% of BMS patients (*n* = 49) had taste disorders [23]. Of 276 BMS patients, 74 (26.8%) were reported to have hypozincemia and zinc replacement therapy, which improved the symptoms of BMS [14]. Zinc is essential for many metabolic and enzymatic functions [26]. These findings suggest that zinc measurement should be performed at least once in patients with BMS.

The number of patients with taste disorders in Japan is increasing [27]. In 2003, it was reported that the number of patients with taste disorders in Japan was 240,000 per year, having a 1.8-fold increase from a survey in 1990 [27]. One of the reasons for this is the annual increase in the elderly population in the country [28].

The death of the mother due to terminal cancer triggered the onset of depression in the patient in this report. Subsequently, the death of her eldest son due to illness and the fact that she was currently living alone may have aggravated her depression and led to the development of BMS. The patient had not received any explanation regarding the pathogenesis of the tongue condition despite visiting several medical institutions over a period of 4–5 months. Knowledge and understanding of BMS in medical and dental clinics are necessary in order to be able to treat this condition at an early stage.

We had previously reported a case in which an oral care gel was effectively used in a patient with BMS [19]. In this case of the current study, oral care gel was used as an adjunct to relieve the pain in the tongue. Our findings indicate that, although the mechanism remains unknown, the application of an oral care gel might be effective in treating burning sensation in the mouth.

As of May 31, 2021, the number of people infected with SARS-CoV-2 in Japan was 746,745 and the number of deaths was 13,060. It was pointed out that the impact of COVID-19 on people is immeasurable, with significant psychological, social, and economic damage leading to increased mental illness, suicide, DV, and abuse of children and the elderly [29]. The number of BMS patients is expected to increase with the outbreak of SARS-CoV-2.

In conclusion, in this study, the adjunctive use of an oral care gel was effective in improving the subjective symptoms and QOL of the patient with burning sensation in the mouth. When patients with burning sensation in the mouth visit the dental clinic, it is important to check for the presence of hypozincemia and a history of depression and to provide collaborative treatment with the family physician or specialist. During the COVID-19 pandemic, dentists need to provide medical interventions and emotional support while listening to the patient's complaints and concerns.

## Figures and Tables

**Table 1:** Medications that the patient was taking at the time of the first visit.

Generic names	Japan trade names	Treatment in this case	Side effect		
			Oropharyngeal discomfort (yes/no)	Dry mouth (yes/no)	Taste disorders (yes/no)
Irbesartan and amlodipine besilate	ILUAMIX combination tablets	Hypertension	No	Yes (frequency: unknown)	Yes (frequency: unknown)
Bepridil hydrochloride hydrate	BEPRICOR tablets	Angina pectoris, and idiopathic atrial fibrillation	No	Yes (frequency: less than 0.1–0.5%)	No
Dabigatran etexilate methanesulfonate	PRAZAXA capsules	Idiopathic atrial fibrillation	Yes (frequency: less than 1%)	No	No
Limaprost alfadex	Limaprost alfadex	Cervical and lumbar stenosis	No	Yes (frequency: less than 0.1%)	Yes (frequency: less than 0.1%)
Mecobalamin	Methycobal	Cervical and lumbar stenosis	No	No	No
Tocopherol acetate	Juvela	Cervical and lumbar stenosis	No	No	No

**Table 2:** VAS score before the dental visit and during REFRECARE-H application.

Subjective symptoms	Before visit (before application)	After visit (during of application)
	April 2019	Mean ± SD
Dry mouth (mm)	100	5.76 ± 10.29
Breath odor (mm)	78	1.07 ± 4.06
Refreshing mouth (mm)	83	7.01 ± 10.40
Oral pain during rest (mm)	60	6.14 ± 4.90
Oral pain at a mealtime (mm)	54	0.46 ± 3.92
Taste disorder (mm)	79	0.94 ± 4.33
Loss of appetite (mm)	73	12.17 ± 19.78
Sleep disorder (mm)	75	48.17 ± 24.07
Depressive moodiness (mm)	89	16.92 ± 24.53
Jitteriness (mm)	63	10.92 ± 16.75

SD: standard deviation.

**Table 3:** Summary of laboratory data.

	Normal range	213 days after the first visit	1 year and 201 days after the first visit	1 year and 243 days after the first visit	1 year and 304 days after the first visit
Date		April 6, 2020	March 26, 2021	May 7, 2021	July 7, 2021
Age		75 years old	76 years old	76 years old	76 years old
Taste disorder by taste test		Positive	Negative	Negative	Negative
Dry mouth using an oral moisture-checking device		Negative	Negative	Negative	Negative
White blood cell (*μ*L)	3,500–9,200	5,400	4,400	5,100	2,410
Red blood cell (10^4^/*μ*L)	370–490	482	460	496	467
Hemoglobin (g/dL)	11.5–15.5	13.9	13.3	14.0	13.3
Hematocrit (%)	35.0–45.0	41.0	40.8	43.6	41.1
Platelet count (10^4^/*μ*L)	10.0–40.0	20.3	24.4	28.7	20.6
Fasting blood glucose (mg/dL)	70–110	Unknown	Unknown	Unknown	90
HbA1c (%)	4.6–6.2	5.8	Unknown	Unknown	Unknown
Total protein (g/dL)	6.7–8.3	Unknown	Unknown	6.8	6.1
Albumin (g/dL)	4.0–5.0	Unknown	Unknown	4.4	4.1
Total bilirubin (mg/dL)	0.3–1.2	Unknown	Unknown	0.4	0.6
ALP (U/L)	38–113	Unknown	Unknown	101	67
LDH (U/L)	124–222	Unknown	189	226	165
AST (U/L)	13–33	17	16	20	18
ALT (U/L)	6–30	16	14	16	14
*γ*-GTP (U/L)	10–47	15	18	16	15
Total cholesterol (mg/dL)	128–219	Unknown	239	225	226
HDL, mg/dL	40–96	48	Unknown	Unknown	36
LDL (mg/dL)	80–139	162	117	Unknown	137
Triglyceride (mg/dL)	30–149	208	501	Unknown	133
CK (U/L)	45–163	Unknown	80	Unknown	55
BUN (mg/dL)	8.0–20.0	Unknown	17.1	12.8	12.5
Creatinine (mg/dL)	0.40–0.70	0.70	0.68	0.76	0.75
Na (mEq/L)	138–146	Unknown	140	144	140
K (mEq/L)	3.6–4.9	Unknown	4.3	4.0	4.2
Cl (mEq/L)	99–109	Unknown	102	104	105
Ca (mg/dL)	8.7–10.3	Unknown	9.1	Unknown	8.9
Zinc (*μ*g/dL)	80–130	Unknown	67	70	64
Copper (*μ*g/dL)	66–130	Unknown	Unknown	147	Unknown
FT4 (ng/dL)	0.90–1.70	Unknown	1.03	Unknown	Unknown
TSH (*μ*IU/mL)	0.500-5.000	Unknown	3.440	Unknown	Unknown

ALP: alkaline phosphatase; LDH: lactic dehydrogenase; AST: aspartate aminotransferase; ALT: alanine aminotransferase; *γ*-GTP: gamma-glutamyl transpeptidase; HDL: high-density lipoprotein; LDL: low-density lipoprotein; FT4: free thyroxine 4; TSH: thyroid-stimulating hormone.

## Data Availability

All data generated or analyzed during this study are included in this published article.

## Abbreviations

BMS: Burning mouth syndrome
SARS-CoV-2: Severe acute respiratory syndrome coronavirus 2
COVID-19: Coronavirus disease 2019
QOL: Quality of life
DV: Domestic violence
VAS: Visual analogue scale.
